# Biodegradable Hydrophilic Polyurethane PEGU25 Loading Antimicrobial Peptide Bmap-28: A Sustained-release Membrane Able to Inhibit Bacterial Biofilm Formation in Vitro

**DOI:** 10.1038/srep08634

**Published:** 2015-03-02

**Authors:** Jianzhong Wang, Qinyu Liu, Ye Tian, Zhongyu Jian, Hong Li, Kunjie Wang

**Affiliations:** 1 Department of Urology of West China Hospital, Sichuan University, Chengdu. 610041, China; 2 Department of Urology of the First Affiliated Hospital of Anhui Medical University, Hefei. 230032, China

## Abstract

Catheter-related infection makes up a large part of hospital infection and contributes 80% to all nosocomial urological infection, costing hundreds of millions dollar per year for treatment. Biodegradable hydrophilic material incorporating antibiotic substance is a promising way to prevent catheter-related infection. And antimicrobial peptide seems an optimal drug for its desirable antibiotic effect. In the current research, we produced a new kind of antibiotic material by incorporating antimicrobial peptide Bmap-28 with polyurethane PEGU25 and tested its effect on Proteus mirabilis in vitro. Compared with the control group, PEGU25 membrane incorporating Bmap-28 had a significant lower bacteria load after co-cultured with the Proteus mirabilis. And its antibiotic effect could be observed throughout the whole 7-day test. Also the Bmap-28 membrane could delay catheter obstruction caused by encrustation. Our findings reveal that PEGU25 incorporating Bmap-28 can well inhibit bacterial biofilm formation of common pathogens for catheter-related urinary tract infection in vitro, which makes it a promising antibiotic material for medical tubes for urology.

The consumption of medical tubes for urology, urinary catheter and ureteral stent tube included, is over 100 million per year around the world. The usage of medical tubes leads to more than 1 million cases of urological infection and makes it the most common hospital infection. Catheter-related infection contributes 80% to all nosocomial urological infection^1^. The cost on the therapy for catheter-related urological infection exceeds 450 million dollars every year in the USA^2^. Catheter-associated urinary tract infections (CAUTIs) includes urethritis, cystitis and pyelonephritis and can lead to bacteremia, septicemia or even death^3^ Infection has already severely affected the broad usage of biological materials in clinic and drawn extensive attention recently. Developing high-quality antibiosis materials for clinical use now becomes more and more necessary.

Formation of biofilm on the surface of biological materials is a key part for bacterial infection as well as bacterial drug resistance^4^. Hence prevention of biofilm formation is one of the mainstream research targets at present. Approaches to this target include anti-bacterial adhesion, inactivating adhered bacteria and a combination of the two mentioned above. Anti-bacteria adhesion alone has limited effect on inhibiting biofilm formation and some traditional anti-adhesion materials, such as polyethylene glycol, are unable to prevent bacteria adhering perfectly^5^,^6^. Then an idea of combining medical material with bactericidal substance comes out.

Polyurethane material has excellent physical properties and good biocompatibility and has already been used as implanted biomaterial for about 4 decades. One of the most prominent features of polyurethane is the flexibility of its molecular design. This feature allows polyurethane able to be modified in various ways to meet different clinical needs, biodegradable material included. Santerre et al.^7^ believed that biodegradable polyurethane could be an ideal drug carrier and this perspective was confirmed by Sivak et al. In 2008, Sivak et al.^8^ successfully developed a lysinediisocyanate (LDI) –glycerol polyurethane implant which was able to achieve controlled release of DB-67, and proved its satisfactory anti-tumor activity in vitro.

Antimicrobial peptides own potent and broad-spectrum antimicrobial ability with many desirable properties. They have spectrum of antimicrobial, antiviruses, anti-fungi and anti-parasites together with effects on anti-tumor, immune regulation, promoting wound healing, and so on. In addition, most bacteria have few or none resistance to antimicrobial peptides. The positive charge and amphiphilic nature of antimicrobial peptides (AMPs) provide the peptides the ability to interact with bacterial membranes non-specifically^9^. Membranes or some internal target being structurally modified, bacteria are directly killed by the peptides^10^. Innate immune modulation also contributes to antimicrobial ability of the peptides.

As a result, we became interested in whether biodegradable polyurethane material loading antimicrobial peptides is capable to inhibit the formation of bacteria biofilm. We carried out the following experiments to answer this question.

## Results

After the test of drug sensitive (seen in Table 1), we chose Bmap-28 as our target AMP loaded on membrane. Cell toxicity test showed the highest concentration at toxicity of level 0 of Bmap-28 was higher that of ciprofloxacin and quaternary ammonium salt (Table 2). PEGU25 showed no influence on bladder smooth muscle cell and fibroblast proliferation even at a concentration of 256 μg/ml compared with control group (p = 0.902) (Table 3).

**Figure 1:**
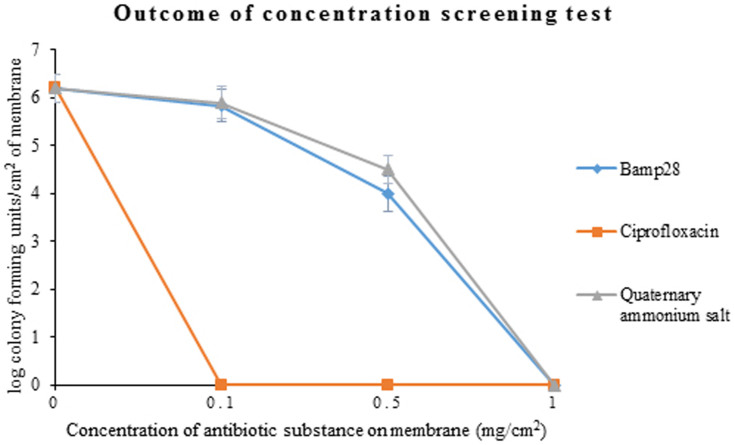
The colony forming units/cm^2^ of different membranes after being co-cultured with Proteus mirabilis (ATCC12453) for 48 h. The original point “0” of y axis means no or very little bacteria load unable to be detected by colony counting.

**Figure 2:**
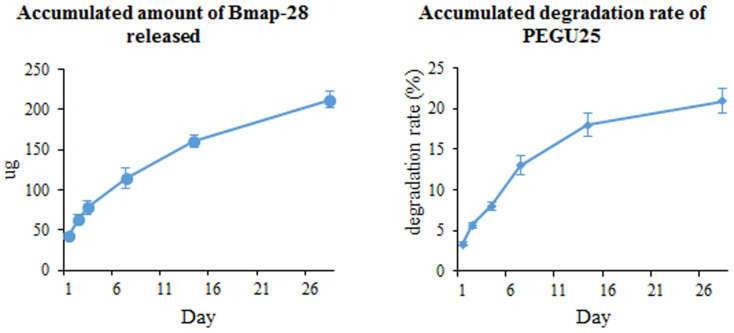
The accumulated amount of Bmap-28 released and accumulated degradation rate of PEGU25 of a 1 cm^2^ membrane with 1 mg Bmap-28.

Then after a 48 h co-culture, no-biofilm status was for the first time observed when Bmap-28 concentration reached 1 mg/cm^2^ on the membrane (Figure 1). PEGU25 degeneration rate and Bmap-28 release rate test showed daily degeneration of PEGU25 was gradually slow down so as the daily release rate of drug (Figure 2). The accumulated degeneration rate of was about 3.3% on the first day and finally reached about 21% on the last day of our test, namely day28, releasing 42 μg and 212 μg Bmap-28 in total respectively. Rectilinear correlation existed between PEGU25 degeneration and drug release (R = 0.998, p < 0.001).

As a result we decided to make PEGU25 membrane loading 1 mg/cm^2^ Bmap-28 as our test membrane in the subsequent 2 different antibiotic ability tests, P. mirabilis as target pathogen. Every test mentioned above was repeated for 3 times and the outcomes were as followed.

### Colony counting (static urine test)

**Figure 3:**
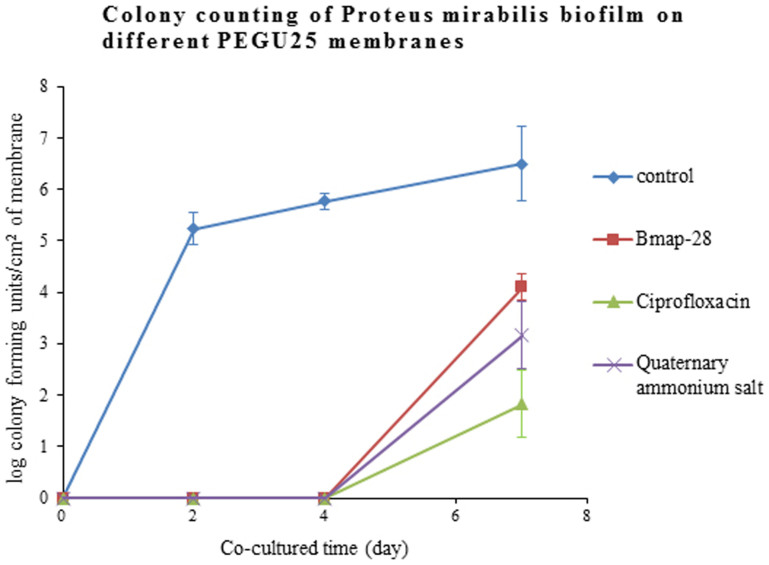
After being co-cultured with Proteus mirabilis (ATCC12453) for 2, 4, and 7days, the colony forming units/cm2 of membrane (log) was 5.20 ± 0.25, 5.78 ± 0.12 and 6.48 ± 0.59 on blank membranes; 0, 0 and 4.12 ± 0.22 on Bmap-28 membranes; 0, 0, 3.23 ± 0.54 on quaternary ammonium salt membranes; 0, 0 and 3.23 ± 0.54 on ciprofloxacin membranes, respectively. Every value mentioned above was the mean value calculated from 3 replicated experiments. The original point “0” of y axis means no or very little bacteria load unable to be detected by colony counting.

Compared with the control group, membranes loading Bmap-28, ciprofloxacin and quaternary ammonium salt all had a significantly lower colony counting outcome (p < 0.001) after being incubated with Proteus mirabilis. Living bacteria counting on Bmap-28 membrane was comparable to that on quaternary ammonium salt (p = 0.167) but a little higher than ciprofloxacin (p = 0.006). (Figure 3)

### FCM and SEM outcomes

**Figure 4:**
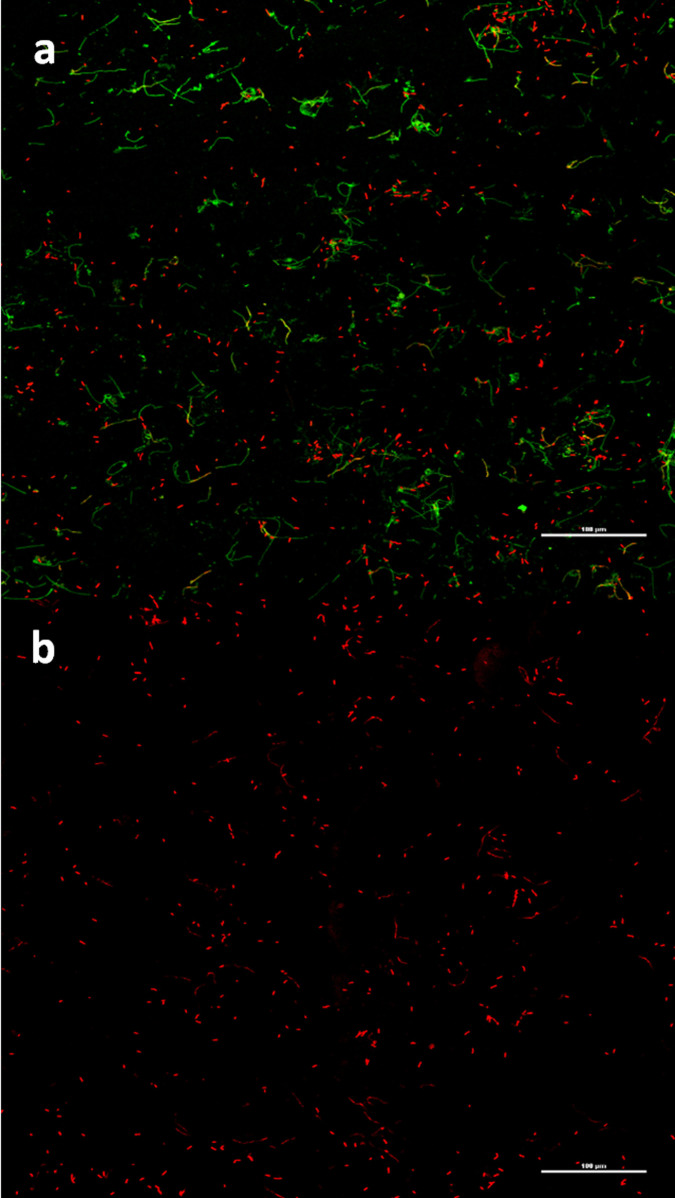
PEGU25 membranes under a fluorescence confocal microscopy (×200) with PI/cyto9 fluorescence staining. (a–b). Proteus mirabilis biofilm on a control membrane (a) and Bmap-28 membrane (b) on day4. Living bacteria present green while the dead one present red.

With PI/cyto9 fluorescence staining, the membranes were put under a FCM to analyze the living status of bacteria adhered on them. Living bacteria were dyed green with cyto9 while the dead were red with PI under the microscope. FCM revealed that on PEGU25 membranes loading antibiotic substances, Bmap-28 included, adhered biofilms were of much smaller area compared with that on the blank membrane. In addition, the majority of bacteria planting on membranes with antibiotic substances were dead. (Figure 4)

**Figure 5:**
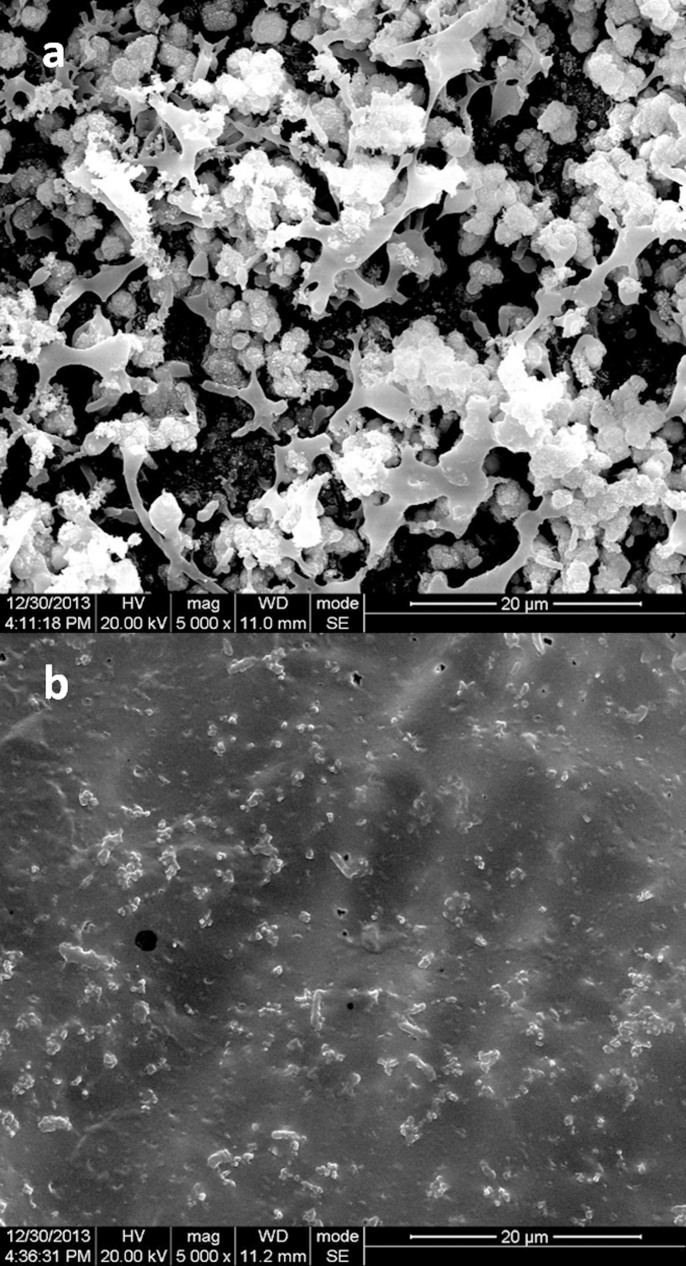
PEGU25 membranes under a scanning electron microscope (×5000). (a–b). Proteus mirabilis biofilm on a control membrane (a) and Bmap-28 membrane (b) on day2. Component analysis revealed that calcium and magnesium phosphate were main component of these biofilms.

SEM showed the detail of these bacteria biofilms. Blank membranes had much thicker biofilms, in which bacteria planted, than any other membranes in both Proteus mirabilis test (Figure 5). Encrustations were noticed in these biofilms and the components of them were similar. Component analysis revealed that calcium and magnesium phosphate made up the majority of the Proteus mirabilis biofilm encrustation.

### Artificial simulated bladder outcomes

Outcomes of tests with flowing artificial urine in an artificial simulated bladder were showed in Table 4. The experiment groups prolonged obstruction time from 21.37 ± 2.11 hours (control group) to at least 64 hours. Also we noticed that the pH of residual urine had significant difference before and after obstruction (6.28 ± 0.11 vs 8.48 ± 0.24, p < 0.001). When the catheters were obstructed, the level of bacteria load (log) was obviously higher that on the membrane (8.72 ± 0.64 vs 7.85 ± 0.47, p < 0.001).

## Discussion

Pioneer studies have proven that Bmap-28 is a potent antimicrobial peptide which is effective against a large range of bacteria, both Gram-positive and Gram-negative ones included^11^,^12^,^13^.Some results even indicate Bmap-28 are comparably effective against antibiotic-resistant and antibiotic-susceptible isolates, MRSA included^11^. The current study also showed Bmap-28 had acceptable effect on E. coli, S. aureus and P. mirabilis, which are common pathogens in CAUTIs. Bmap-28 is a cow-sourced AMP which belongs to mammalian AMP. AMPs of this kind contain proline and some specific structures in sequence which provides them protease resistance^14^. This allow Bmap-28 to work well in the surrounding filled with proteases, in human body for example, since the peptide can serve their antibiotic function as long as they are not degraded. Though Bmap-28 is not human-derived AMPs, our toxicity test proved it even safer than ciprofloxacin. As a result, we believed Bmap-28 could have a good performance in living body apart from cow. In fact an in-vivo research has already given evidences that catheter-related infection in rat could be prevented with central venous catheters pre-treated with Bmap-28^15^. Another study shows ureteral stents incubated with Bmap-28 before implanted results in reduced biofilm^16^.

In the current study, we combined Bmap-28 on a biodegradable hydrophilic PEGU25 membrane at 1 mg/cm^2^ to create a sustained-release membrane and tested its property. Polyurethane has been proven an ideal drug carrier with its features such as biodegradable, degradation products non-toxic, molecular design flexible and excellent self-assemble ability^17^,^18^,^19^. Polyurethane PEGU25 confirmed these features mentioned above in our pre-test. The toxicity test showed that it had little effect on human cells even at a concentration as high as 256 μg/ml. Meanwhile degeneration and drug-release test suggested that drug was uniformly distributed on the membrane and degeneration of PEGU25 would not affect the structure of Bmap-28. All of these proved it a biomaterial with satisfying bio-security and drug loading capacity.

As for antibiotic ability, outcomes of the current study indicated that Bmap-28, loaded on PEGU25, could had a satisfying antibacterial ability to Proteus mirabilis for at least 7 days. We noticed that Bmap-28 was comparable with quaternary ammonium salt against Proteus mirabilis but less effective than ciprofloxacin to some degree.

Antibiotics, heavy metal, cationic surface-active agents such as quaternary ammonium salt and antimicrobial peptides can all be candidates for bactericidal substance loaded on drug carrier. However, antibiotics, heavy metal and quaternary ammonium salt may have shortages including short effectiveness, narrow antimicrobial spectrum, high cytotoxicity and potential possibility of drug resistance^20^,^21^. Antimicrobial peptides are able to avoid all limitation mentioned above^22^. In the current study, ciprofloxacin appeared to be the most effective antibiotic substance loaded on the PEGU25 membrane. However, considering that long-time local application of antibiotics can lead to drug resistance and secondary infection of other opportunistic pathogen, we don’t recommend it as an ideal candidate incorporated on the catheter.

An interesting finding was that when we refreshed the urine after the first 48 h, though the release of Bmap-28 couldn’t make its concentration remain above MBC because of the slowing down of PEGU25 degeneration, no biofilm was found in the next 48 hours. Scientific researchers have confirmed some human-derived AMPs have the properties of preventing biofilm formation at sub-inhibitory concentrations by assisting the human defense system^23^. We presume Bmap-28 may also have similar function approach. In addition, the biodegradable hydrophilic feature of PEGU25 provides it with an ability to reduce bacteria adhesion, assisting Bmap-28 in preventing biofilm formation. We intend to produce antibiotic catheters or stents in the future by spraying and incubating the tubes with PEGU25 emulsion loading Bmap-28, providing them with biodegradable drug-release coatings. So this phenomenon also inspired us that multilayer spraying with different concentration may be a way to prolong the effective time against slowed degeneration rate.

Our SEM images demonstrated that biofilms of Proteus mirabilis provided living space for bacteria and meanwhile contributed to encrustation which block the catheter. Also it was interesting to notice that the pH of urine had a distinct increase when encrustation formed. Urease is thought to be one of the key parts for encrustation formation. It has the ability to alkalize the urine by hydrolyzing to urea, enhancing the absorption of calcium and magnesium on biofilm^24^. Stickler et al.^25^ believed it was Proteus mirabilis that synthesis urease and the current study confirms this perspective. From the outcomes of artificial simulated bladder test we can conclude that Bmap-28 sustained-release membrane, by inhibiting bacteria proliferation and biofilm formation, can well delay the obstruction in a slightly acidic environment. This indicates Bmap-28 may reach a better result in anti-biofilm-formation with the assistance of acidification of urine.

Another noteworthy phenomenon is that the bacteria load was obviously higher that on the membrane when catheter obstruction happened. We suspect that Bmap-28 membrane in the current study can effectively eliminate the adhered bacteria but slightly less effect on the floating ones since there may exist a concentration gradient of Bmap-28 between the surface and liquid around, which indicates that Bmap-28 membrane alone can be an promising antibiotic material to prevent catheter-related infection but a higher dosage or adjuvant therapy of other medicines is needed when facing existing infection. Further studies are expected to explore the answer.

## Methods

### Materials and equipment

#### Polyurethane emulsion

**Figure 6:**
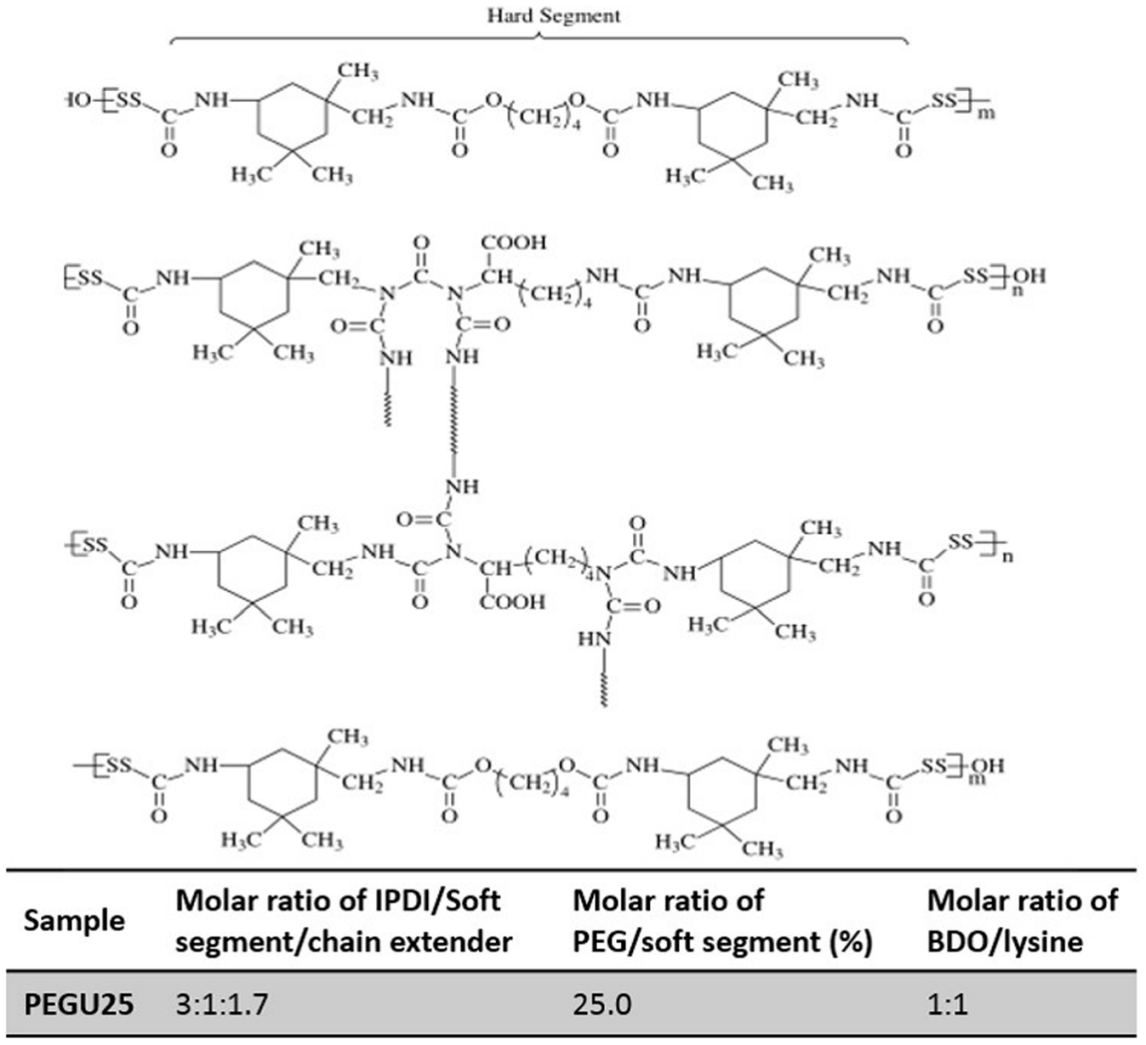
Schematic structure of biodegradable hydrophilic polyurethane PEGU25. n = 1, 2, 3,..., m = 1, 2, 3,..., Soft Segment (SS) is polyethylene glyco (PEG) or PCL (polycaprolactone). 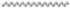 is isophorone diisocyanate (IPDI) or polyurethane (PU) chain segment.

Emulsion of polyurethane PEGU25 (Patent No. ZL 200610022715.2) (Figure 6) was produced and provided by Polymer Research Institute, Sichuan University (Sichuan, China).

#### Antibiotic substance

Bmap-28 (GGLRSLGRKILRAWKKYGPIIVPIIRIG^26^), SgI-29 (HNKQEGRDHDKSKGHFHRVVIHHKGGKAH^27^) and Citropin 1.1 (GLFDVIKKVASVIGGL-NH2^28^) was customized and bought from ChinaPeptides Co., Ltd. (Shanghai, China). The purity of each AMP is over 95%. Ciprofloxacin (Bayer AG, Leverkusen, Germany) and quaternary ammonium salt (purity 97%) were bought from Sheng Technology Co., Ltd (Sichuan, China).

#### Organisms

Proteus mirabilis (ATCC12453) was bought from Bianzhen Biological Technology Co., Ltd.(Nanjing, China). In this study, lyophilized cultures were reconstituted in tryptose broth (LB) for 18 hours (37°C, 220 r/min) then plated on tryptic soy agar and incubated (37°C, for 24–48 hours). Single colonies were picked and transferred to LB and incubated for 18 hours (37°C, for 18 hours). The cultures were adjusted to an optical density of 0.218 for Proteus mirabilis (ATCC12453) at 600 nm in the spectrophotometer, representing 1.0 × 10^8^ CFU/ml. Then the bacterium solution was dilute 10^−1^fold to 1.0 × 10^7^ CFU/ml.

#### Artificial urine

The ingredients of artificial urine were introduced by Griffith et al.^29^ as followed: CaCl_2_ 0.49 g/l, MgCl_2_·6H_2_O 0.65 g/l, NaCl 4.6 g/l, Na_2_SO4 2.3 g/l, sodium citrate 0.65 g/l, sodium oxalate 0.02 g/l, monopotassium phosphate 2.8 g/l, KCl 1.6 g/l, NH_4_Cl 1.0 g/l,urea 25 g/l, gelatine powder 5.0 g/l and tryptic soy broth 1.0 g/l, adjusted to pH 6.1 with NAOH. The artificial urine was filtrated to get rid of bacteria when prepared.

#### Artificial simulated bladder

**Figure 7:**
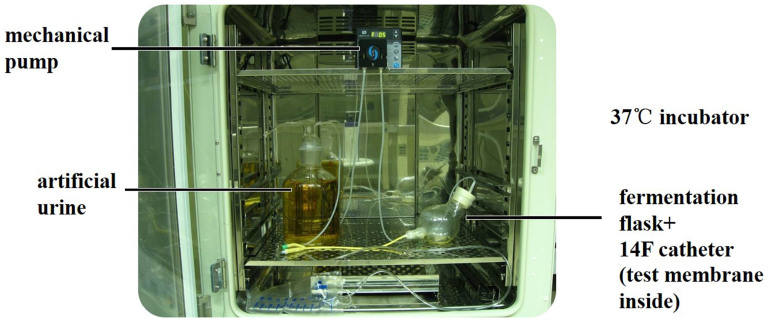
The artificial simulated bladder, artificial urine (on the left) was pumped into the “bladder” (on the right) at a rate of 0.5 ml/min then flowed out through the catheter.

An artificial simulated bladder was a fermentation flask placed in a incubater at 37°C, with a urinary catheter attached to its lower outlet was a urethra. Artificial urine in a sealed glass container was pumped into the simulated bladder at a rate of 0.5 ml/min then flowed out through the catheter. (Figure 7)

Other materials and equipment included Cell counting kit-8 (CCK8), PI/cyto9 fluorochrome, methanol, glutaraldehyde, ultrasonic vibration device, scanning electron microscope (SEM), fluorescence confocal microscopy (FCM), ultraviolet spectrometry photometer, shaking table, incubator and so on.

### Methods

#### Sensitive and toxicity of different antibiotic substance

Briefly, to detect MIC and MBC of different antibacterial substances, constant broth dilution method (128, 64, 32, 16, 8, 4, 2, 1, 0.5, 0.25, 0.125 μg/ml of substance tested) was employed, Bmap-28, SgI-29, Citropin 1.1, ciprofloxacin and quaternary ammonium salt being tested. Colony counting was used to tell MIC and MBC.

As for toxicity test, different concentrations of each tested material were cultured together with human fibroblast L929 cells and bladder smooth muscle cells (SMC), and then detected the cytotoxicity of different material by CCK8 method. Relative growth rate (RGR) over 80% meant toxicity level 0. Selected antibiotic substances and PEGU25 were tested.

#### Preparation of biodegradable hydrophilic polyurethane membrane

Polyurethane PEGU25 emulsion of 150 μl was mixed with Bmap-28 of 0.1, 0.2, 0.5 and 1 mg then transferred in a 48-well plate, the basal area of which was about 1 cm^2^. After that the 48-well plate was placed into an oven at 60°C for 48 hours. The PEGU25 emulsion was dried to form a membrane subsequently. Blank PEGU25 membranes were prepared the same way to perform as the control group.

#### Degeneration, drug release and drug dose screening test

For degeneration rate test, 3 blank PEGU25 membranes (1 cm^2^ each) were placed into 2 ml artificial urine respectively in 37°C constant temperature shaker. Mean value of total weight losing rate was detected on certain day. Artificial urine was refreshed every 48 hours.

Three PEGU25 membranes loading 1 mg/cm^2^ Bmap-28 were employed for drug releasing test. Membrane treated the same way of degeneration rate test. A fluorescence detector was used on certain day to detect fluorescence value (EX peak) and release dose was calculated based on a standard EX peak curve of Bmap-28 established in our pre-test. Artificial urine was refreshed every 48 hours.

PEGU25 membranes loading 0.1, 0.2, 0.5 and 1 mg/cm^2^ Bmap-28 were respectively co-cultured with 0.2 ml bacterium solution (10^7^ cfu/ml, Proteus mirabilis) in 1.8 ml artificial urine at 37°C for 48 h. Colony counting was the way to assess the bacteriostatic ability after bacteria being stripped by shockwave.

#### Test on bacteria biofilm formation with different PEGU25 membranes in artificial urine (static state)

PEGU25 membranes (1 cm^2^ each) loading antibiotic substance (Bmap-28, ciprofloxacin or quaternary ammonium salt, 1 mg/cm^2^) were respectively co-cultured with 0.2 ml bacterium solution (10^7^ cfu/ml, Proteus mirabilis) in 1.8 ml artificial urine at 37°C for 2, 4 and 7 days. Artificial urine was refreshed every 48 hours. As a control, blank membranes were co-cultured with bacteria in the same way. After co-culturing, PEGU25 membranes were gently rinsed to clear floating bacteria. Semi-quantitatively, Immunofluorescence technique (with Live/Dead® Baclight TM Bacterial Viability Kit) was used to detect living bacteria on the PEGU25 membranes and SEM was employed to reveal the status of biofilm. Colony counting was the way to assess the bacteriostatic ability quantitatively after bacteria being stripped by shockwave.

#### Test on bacteria biofilm formation with different PEGU25 membranes in artificial simulated bladder

Together with 10 ml of Proteus mirabilis solution (10^8^ cfu/ml), 5 PEGU25 membranes (loading Bmap-28, ciprofloxacin, quaternary ammonium salt or blank), namely 5 cm^2^ in total, were transferred to an artificial simulated bladder placed in a thermostatic incubator at 37°C. Artificial urine in a sealed glass container was pumped into the simulated bladder at a rate of 0.5 ml/min then flowed out through the catheter attached to the outlet of the simulated bladder, making the remained urine stay stable at around 30 ml. The endpoint of this test was happening of catheter obstruction or test-time over 72 hours. Obstruction time (hour) and pH of residual urine were recorded after endpoint. Colony counting of bacteria adhered to the membrane and colony counting of bacteria floating in the urine were done only when catheter obstruction happened.

#### Statistics

SPSS 20.0 Statistical System was used to analyze the data. All data were presented in the style of mean ± standard deviation (SD). An unpaired Student's-t-test was used for data analysis between two groups. Multivariate analysis of variance (MANOVA) was used for multiple groups and comparisons between every two groups were done with LSD test. Differences were considered statistically significant when p < 0.05.

## Author Contributions

J.W. and Q.L. contributed equally to this work and share the co-first authors. J.W., Q.L. and Y.T. designed all the experiments; J.W., Q.L., Y.T. and Z.J. performed the experiments. J.W. and Q.L. did the statistical analysis. J.W. and Q.L. wrote the manuscript while Y.T., K.W. and H.L. provided suggestions for revision. K.W. obtained the funding. K.W. and H.L. did the supervision job throughout this study.

## Figures and Tables

**Table 1:** MIC and MBC of different antibiotic substances for different bacteria

	E. coli (ATCC25922)		S. aureus (ATCC25923)		P. mirabilis (ATCC12453)	
	MIC (μg/ml)	MBC (μg/ml)	MIC (μg/ml)	MBC (μg/ml)	MIC (μg/ml)	MBC (μg/ml)
CPFX	<0.25	0.4	<0.25	0.3	0.5	1.3
QAS	13.3	21.3	6.7	13.3	16	21.3
Bmap-28	6.7	10.7	8	10.7	10.7	21.3
Sgi-29	10.7	26.7	6.7	21.3	>128	>128
Citropin1.1	42.7	>128	26.7	85.3	>128	>128

*MIC* minimum inhibitory concentration, *MBC* minimal bactericidal concentration, *QAS* quaternary ammonium salt, *CPFX* ciprofloxacin.

**Table 2:** Toxicity of different antibiotic substances on fibroblast (L-929) and SMC of bladder

	Max concentration at toxicity of level 0(μg/ml)	
	L-929	SMC
CPFX	32	32
QAS	32	32
Bmap-28	64	64

*SMC* smooth muscle cell, *QAS* quaternary ammonium salt, *CPFX* ciprofloxacin.

**Table 3:** Absorbance of cell in different PEGU25 concentration groups

μg/ml	0	8	16	32	64	128	256
SMC	1.51 ± 0.12	1.49 ± 0.23	1.49 ± 0.17	1.45 ± 0.20	1.46 ± 0.14	1.45 ± 0.27	1.46 ± 0.32
L-929	1.01 ± 0.25	0.97 ± 0.32	0.98 ± 0.28	0.94 ± 0.35	0.94 ± 0.24	0.95 ± 0.17	0.94 ± 0.20

*SMC* smooth muscle cell, *L-929* fibroblast L-929.

**Table 4:** Outcomes of artificial simulated bladder related tests

Group		OT (h)	pH (NO)	PH (O)	Log (cfu/ml of urine)	Log (cfu/cm2 of membrane)
control	1	21	-	8.7	9.5	8.2
	2	24	-	8.6	8.7	7.9
	3	19	-	8.9	9.7	8.7
QAS	1	>72	6.3	-	-	-
	2	70	-	8.5	8.5	7.8
	3	65	-	8.3	7.7	7.0
CPFX	1	>72	6.1	-	-	-
	2	>72	6.3	-	-	-
	3	68	-	8.4	8.5	7.8
Bmap-18	1	64	-	8.3	8.8	7.7
	2	>72	6.4	-	-	-
	3	68	-	8.1	8.0	7.5

*OT* obstruction time, *NO* non-obstructed, *O* obstructed, *QAS* quaternary ammonium salt, *CPFX* ciprofloxacin.
